# Visando Equidade em Saúde: Por que a Diversidade na Pesquisa Médica Brasileira é Importante?

**DOI:** 10.36660/abc.20240731

**Published:** 2024-12-11

**Authors:** Christina Grüne de Souza e Silva

**Affiliations:** 1 Clínica de Medicina do Exercício Rio de Janeiro RJ Brasil Clínica de Medicina do Exercício – CLINIMEX, Rio de Janeiro, RJ – Brasil; 2 Instituto do Coração Edson Saad da Universidade Federal do Rio de Janeiro Rio de Janeiro RJ Brasil Instituto do Coração Edson Saad da Universidade Federal do Rio de Janeiro (UFRJ), Rio de Janeiro, RJ – Brasil

**Keywords:** Diabetes Mellitus, Obesidade, Fatores de Risco, Vulnerabilidade Social, Políticas Públicas

Há mais de 35 anos, os cidadãos brasileiros conquistaram seu direito constitucional à saúde.^1^ Orientados pelos princípios fundamentais da universalidade e da equidade, o acesso à saúde e as ações oferecidas pelo Sistema Único de Saúde (SUS) são, desde sempre, responsabilidades do Estado e devem ser, portanto, garantidas por políticas econômicas e sociais.

Desde sua criação, o SUS tem experimentado melhorias significativas na prevenção de doenças e promoção da saúde.^2^ Uma queda drástica na mortalidade infantil e um aumento significativo na expectativa de vida são alguns dos principais resultados que refletem a expansão do acesso da população brasileira a cuidados de saúde abrangentes. No entanto, as disparidades entre regiões geográficas e grupos de renda persistem, especialmente para populações vulneráveis, como os quilombolas.

Os quilombolas são pessoas predominantemente negras que vivem majoritariamente em comunidades rurais formadas por descendentes de africanos escravizados que se definem a partir de fortes vínculos com a terra, parentesco, território e ancestralidade e com tradições e práticas culturais específicas.^3^ Embora sua existência e direitos tenham sido reconhecidos em 1988 pela Constituição Brasileira, foi somente em 2022 que o Censo Brasileiro investigou a população quilombola.^4^ Informações sobre suas características demográficas, geográficas e socioeconômicas foram obtidas pela primeira vez, prática fundamental para reduzir sua invisibilidade e orientar a construção de políticas públicas que atendam às suas necessidades específicas. No entanto, e não surpreendentemente, ainda há escassez de estudos sobre o estado de saúde dos quilombolas.

Visando aprofundar o conhecimento sobre o tema, Santana et al.^5^ realizaram um estudo para descrever a prevalência da "diabesidade" e sua associação com fatores de risco isolados ou combinados. A diabesidade é um fenótipo constituído pela coexistência de obesidade e diabetes mellitus tipo 2,^6^ duas epidemias modernas que não mostram sinais de diminuição de sua prevalência em um futuro próximo e que são grandes contribuintes para doenças cardiovasculares, a principal causa de morte no mundo.^7^ Mais de 300 adultos de meia-idade e idosos (53% mulheres, média de idade de 61 anos) da microrregião de Guanambi, Bahia, foram entrevistados sobre consumo de tabaco e/ou álcool, dieta, níveis de atividade física, diagnóstico prévio de diabetes e tiveram sua circunferência da cintura medida para definir a presença de obesidade. Do total de 320 quilombolas incluídos, 18% tinham diabesidade e, curiosamente, não foi encontrada associação entre cada fator de risco relacionado ao estilo de vida e a presença de diabesidade quando ajustado para sexo, idade, nível educacional, estado civil e ocupação. Entretanto, a prevalência observada de cada um dos fatores de risco chamou a atenção. Quando comparados aos últimos resultados nacionais em indivíduos com faixa etária semelhante (55 a 64 anos) obtidos no VIGITEL, inquérito telefônico sobre doenças crônicas e fatores de risco no Brasil,^8^ a comunidade quilombola estudada por Santana et al.^5^ apresentou maior prevalência de consumo de tabaco (48% vs. 10%), álcool (26% vs. 11%) e frutas e vegetais (58% vs. 37%). Por outro lado, os quilombolas apresentaram menor prevalência de inatividade física (24% vs. 49%). Além disso, a prevalência segundo sexo foi diferente entre os quilombolas estudados e os resultados apresentados no VIGITEL. Entre os quilombolas de meia-idade e mais velhos, os homens apresentaram maior consumo de tabaco (67% vs. 27%, p<0,01) e álcool (44% vs. 8%, p<0,01) em comparação às mulheres, mas consumo semelhante de frutas e vegetais (56% vs. 62%, respectivamente, p=0,26) e taxas semelhantes de inatividade física (20% vs. 18%, respectivamente, p=0,59). Por sua vez, nacionalmente, homens e mulheres de 55 a 64 anos apresentaram prevalência semelhante de consumo de tabaco (10%), bebidas alcoólicas foram consumidas por uma porcentagem duas vezes maior de homens do que de mulheres (16% vs. 8%), e uma taxa maior de mulheres eram fisicamente inativas (54% vs. 43%). Deve-se destacar, no entanto, que os critérios usados para definir o consumo de tabaco, álcool e frutas e vegetais diferiram ligeiramente entre esses estudos e, portanto, essa comparação direta deve ser vista com cautela. Por fim, e embora, ao melhor do meu conhecimento, não haja dados disponíveis sobre a prevalência da ocorrência simultânea de diabetes tipo 2 e obesidade no Brasil, 22% da população brasileira com idade entre 55 e 64 anos (23% homens e 22% mulheres) autodeclarou ter tido diagnóstico prévio de diabetes, e 26% (24% os homens e 28% das mulheres) relataram ser obesos.^8^ Como se estima que cerca de 50% da carga de diabetes tipo 2 seja atribuída à obesidade,^9^ pode-se especular que a prevalência de diabesidade observada nos quilombolas pode ser maior do que na população geral brasileira.

Como grande parte do mecanismo de prevenção de doenças cardiovasculares e outras doenças crônicas é por meio da modificação de fatores de risco,^10^ conhecer sua prevalência e monitorar o seu progresso é essencial para orientar sobre onde os esforços fornecidos por políticas públicas estão funcionando ou são atualmente insuficientes e precisam ser revisados. Portanto, ao fornecer informações de saúde sobre fatores de risco relacionados ao estilo de vida e a prevalência de diabesidade em quilombolas de meia-idade e mais velhos, o estudo conduzido por Santana et al.^5^ nos ajuda a entender melhor as necessidades de saúde dessa população vulnerável e dar um passo em direção a um desafio contínuo para reduzir as disparidades de saúde e alcançar a equidade em saúde.
