# Supplementary material for: Visando Equidade em Saúde: Por que a Diversidade na Pesquisa Médica Brasileira é Importante?
Source: Arq Bras Cardiol. 2024 Dec 11;121(11):e20240731. [Article in Portuguese] doi: 10.36660/abc.20240731 (PMC11634316; doi:10.36660/abc.20240731)
Supplement: Translation

# Translation: Aiming for Health Equity: Why Does Diversity in Brazilian Medical Research Matter?

**DOI:** 10.36660/abc.20240731i

**Authors:** Christina Grüne de Souza e Silva

**Affiliations:** 1 Rio de Janeiro RJ Brazil Clínica de Medicina do Exercício – CLINIMEX, Rio de Janeiro, RJ – Brazil; 2 Rio de Janeiro RJ Brazil Instituto do Coração Edson Saad da Universidade Federal do Rio de Janeiro (UFRJ), Rio de Janeiro, RJ – Brazil

**Keywords:** Diabetes Mellitus, Obesity, Risk Factors, Social Vulnerability, Health Policy

Over 35 years ago, Brazilian citizens earned their constitutional right to health.^1^ Guided by the fundamental principles of universality and equity, health access and actions provided by the Brazilian Unified Health System (Sistema Único de Saúde – SUS) are ever-since responsibilities of the State and should be, therefore, guaranteed by economic and social policies.

Since its creation, SUS has experienced significant improvements in disease prevention and health promotion.^2^ A dramatic drop in infant mortality and a significant increase in life expectancy are a couple of major outcomes that reflect the expansion in Brazilians’ population access to comprehensive health care. However, disparities across geographical regions and income groups persist, especially for vulnerable populations such as the quilombolas.

Quilombolas are predominantly black people who live mostly in rural communities formed by descendants of enslaved Africans who define themselves based on strong bonds with land, kinship, territory, and ancestry and with specific traditions and cultural practices.^3^ Although their existence and rights were recognized in 1988 by the Brazilian Constitution, it was only in 2022 that the Brazilian Census investigated the quilombola population.^4^ Information about its demographic, geographic, and socioeconomic characteristics were obtained for the first time, a fundamental practice to reduce their invisibility and to guide the construction of public policies that meet their specific needs. However, and not surprisingly, there is still a scarcity of studies regarding the quilombolas’ health status.

Aiming to deepen our knowledge upon this issue, Santana et al.^5^ conducted a study to describe the prevalence of "diabesity" and its association with risk factors either in isolation or in combination. Diabesity is a phenotype constituted by the coexistence of obesity and type 2 diabetes mellitus,^6^ two modern epidemics that show no signs of decreasing their prevalence in the near future and that are major contributors to cardiovascular disease, the leading cause of death worldwide.^7^ Over 300 middle-aged and older adults (53% female, mean age of 61 years) from the microregion of Guanambi, Bahia, were interviewed regarding tobacco and/or alcohol consumption, diet, levels of physical activity, previous diagnosis of diabetes, and had their waist circumference measured to define the presence of obesity. From the total of 320 quilombolas included, 18% had diabesity, and, interestingly, no association was found between each lifestyle-related risk factor and the presence of diabesity when adjusted for sex, age, educational level, marital status, and occupation. However, the prevalence observed of each of the risk factors called to attention. When compared to the latest national results in individuals with a similar age range (55 to 64 years old) obtained from VIGITEL, a telephone survey concerning chronic diseases and risk factors in Brazil,^8^ the quilombola community studied by Santana et al.^5^ had a higher prevalence of tobacco (48% vs. 10%), alcohol (26% vs. 11%) and fruits and vegetables (58% vs. 37%) consumption. On the other hand, the quilombolas had a lower prevalence of physical inactivity (24% vs. 49%). Moreover, prevalence according to sex was different between the quilombolas studied and the results presented in VIGITEL. Within the middle-aged and older quilombolas, men had higher consumption of tobacco (67% vs. 27%, p<0.01) and alcohol (44% vs. 8%, p<0.01) compared to women, but similar consumption of fruits and vegetables (56% vs. 62%, respectively, p=0.26), and similar rates of physical inactivity (20% vs. 18%, respectively, p=0.59). In turn, nationally, men and women aged 55 to 64 years old had similar prevalence of tobacco consumption (10%), alcoholic beverages were consumed by a percentage twice as high of men than women (16% vs. 8%), and a higher rate of women were physically inactive (54% vs. 43%). It should be highlighted, however, that the criteria used to define tobacco, alcohol, and fruit and vegetable consumption differed slightly between these studies and, therefore, this direct comparison should be viewed with caution. Finally, and although, to the best of my knowledge, no data is available regarding the prevalence of the simultaneous occurrence of type 2 diabetes and obesity in Brazil, 22% of the Brazilian population aged 55 to 64 years old (23% men and 22% women) self-declared having had a previous diagnosis of diabetes, and 26% (24% men and 28% women) reported being obese.^8^ As it is estimated that around 50% of type 2 diabetes burden is attributable to obesity,^9^ one may speculate that the prevalence of diabesity observed in the quilombolas might be higher than nationwide.

As much of the mechanism for cardiovascular and other chronic disease prevention is through the modification of risk factors,^10^ knowing its prevalence and further tracking its progress is essential to give insights into where efforts provided by public policies are working or are currently insufficient and need to be reviewed. Therefore, by providing health information about lifestyle-related risk factors and the prevalence of diabesity in middle-aged and older quilombolas, the study conducted by Santana et al.^5^ helps us better understand the health needs of this vulnerable population and take a step towards an ongoing challenge to reduce health disparities and achieve health equity.
